# Comparison of general and cardiac care-specific indices of spatial access in Australia

**DOI:** 10.1371/journal.pone.0219959

**Published:** 2019-07-25

**Authors:** Vincent Lawrence Versace, Neil T. Coffee, Julie Franzon, Dorothy Turner, Jarrod Lange, Danielle Taylor, Robyn Clark

**Affiliations:** 1 Deakin Rural Health, School of Medicine, Deakin University, Geelong, Victoria, Australia; 2 National Centre for Farmer Health, Western District Health Service, Hamilton, Victoria, Australia; 3 College of Nursing and Health Sciences, Flinders University, Adelaide, South Australia, Australia; 4 Centre for Research and Action in Public Health (CeRAPH), University of Canberra, Canberra, ACT, Australia; 5 Spatial Sciences Group, School of Biological Sciences, The University of Adelaide, Adelaide, South Australia, Australia; 6 Hugo Centre for Migration and Population Research, The University of Adelaide, Adelaide, South Australia, Australia; 7 Basil Hetzel Institute for Translational Health Research, Discipline of Medicine, The University of Adelaide, Adelaide, South Australia, Australia; University of Zurich, SWITZERLAND

## Abstract

**Objective:**

To identity differences between a general access index (Accessibility/ Remoteness Index of Australia; ARIA+) and a specific acute and aftercare cardiac services access index (Cardiac ARIA).

**Research design and methods:**

Exploratory descriptive design. ARIA+ (2011) and Cardiac ARIA (2010) were compared using cross-tabulations (chi-square test for independence) and map visualisations. All Australian locations with ARIA+ and Cardiac ARIA values were included in the analysis (n = 20,223). The unit of analysis was Australian locations.

**Results:**

Of the 20,223 locations, 2757 (14% of total) had the highest level of acute cardiac access coupled with the highest level of general access. There were 1029 locations with the poorest access (5% of total). Approximately two thirds of locations in Australia were classed as having the highest level of cardiac aftercare. Locations in Major Cities, Inner Regional Australia, and Outer Regional Australia accounted for approximately 98% of this category. There were significant associations between ARIA+ and Cardiac ARIA acute (χ^2^ = 25250.73, df = 28, p<0.001, Cramer’s V = 0.559, p<0.001) and Cardiac ARIA aftercare (χ^2^ = 17204.38, df = 16, Cramer’s V = 0.461, p<0.001).

**Conclusions:**

Although there were significant associations between the indices, ARIA+ and Cardiac ARIA are not interchangeable. Systematic differences were apparent which can be attributed largely to the underlying specificity of the Cardiac ARIA (a time critical index that uses distance to the service of interest) compared to general accessibility quantified by the ARIA+ model (an index that uses distance to population centre). It is where the differences are located geographically that have a tangible impact upon the communities in these locations–i.e. peri-urban areas of the major capital cities, and around the more remote regional centres. There is a strong case for specific access models to be developed and updated to assist with efficient deployment of resources and targeted service provision. The reasoning behind the differences highlighted will be generalisable to any comparison between general and service-specific access models.

## Introduction

Health geography seeks to examine the interactions between people’s health and the environment,[1] with research in this field examining concepts such as the patterns, risk, and spread of disease, [2,3,4] and also the planning and provision of the health workforce and services.[5] Relevant to the latter example is the concept of locational disadvantage, with the impact of remoteness and accessibility to health services well established in the literature. [6,7,8,9]

Access can be described as five specific dimensions.[10] These can be grouped into aspatial dimensions (1. Affordability–related to healthcare cost, 2. acceptability–related to cultural considerations, and 3. accommodation–related to communication effectiveness), and spatial dimensions (4. availability–related to capacity to deliver the service, and 5. accessibility–related to travel cost between the provider and the patient).[11] Indeed, it is spatial accessibility, or physical access, to health services that is a persistent challenge in many settings–even in jurisdictions where policies strive for universal access. In Canada, a combination of uneven population and healthcare facilities influences access to services.[12,13] In the United States, where there is not universal healthcare, sparsely populated landscapes and vast distances lead to health disparities.[14] The consistency between these two examples and the Australia experience is that of the underlying geography and population distribution. Despite having one of the highest life expectancies in the world, and a universal healthcare system, the mortality rates and the burden of disease is heavily influenced by where people live–those in rural and remote areas typically have poorer health status than their metropolitan counterparts.[15]

In Australia, the nationally accepted standard for measuring accessibility is the Accessibility/Remoteness Index of Australia (ARIA), developed at the University of Adelaide in 1998. Since development (including updates, namely Accessibility/Remoteness Index of Australia Plus, ARIA+), there have been many publications applying ARIA+ to describe associations with health or health-related issues, (e.g., access to general practitioners, health service utilisation, cancer survival, health workforce retention, and indigenous cancer diagnosis and treatment).[16,17,18,19,20] A key aspect of these papers is the modelling of geographic access or remoteness.

These analyses use maps as the primary output, as spatial visualisations are more easily interpreted–yet information reported in each map depends implicitly on the underlying assumptions and modelling methods. Spatial methodologies and maps may not be as easily interpreted by a reader with a non-spatial background and, as such, modelling limitations may be overlooked.[21] Measuring geographic accessibility is fundamental to understanding how the impact of distance on access to important services is distributed across Australia, especially if we aspire to deliver equity in service provision. Australia is a vast landmass, and while it is generally understood that large regions are sparsely populated, without detailed geographic accessibility models, understanding where this is problematic remains generally, but not specifically known. Accessibility measures provide a useful way to identify inequalities in accessibility, report data, examine relationships between accessibility and other factors, and monitor changes in accessibility levels as a result of the introduction or removal of different services.[22] As ARIA+ is the main accessibility index used for Australian research, it is important to understand how a specific service model compares with a general model, as the model applied can have implications for clinical, social, and political dimensions. This paper is focussed on the importance of understanding the geographic dimension (i.e. physical access) as this is first required before other dimensions play a role in accessing services. Therefore, the aim of this research was to compare the underlying conceptual similarities and differences between the more general ARIA+ (2011) and the service specific Cardiac ARIA index (2010) and identify locations where the levels of accessibility substantially differ across models to highlight the appropriate use of geographically modelled data.

## Methods

### Design

This study used an exploratory descriptive design.

### The Accessibility/Remoteness Index of Australia (ARIA)

In the mid-1990s the then Commonwealth Department of Health and Ageing worked with the National Key Centre for Teaching and Research into the Social Applications of Geographic Information Systems (GISCA, now the Hugo Centre for Migration and Population Research) at the University of Adelaide to construct a model of accessibility and remoteness for Australia.[23] The aim of this exercise was to provide a simple, nationally consistent model that would quantify access along a continuum from highly accessible to very remote. The resultant ARIA+ has become the standard for access measurement in Australia and the basis for the Australian Bureau of Statistics (ABS) remoteness classification.[24] The ARIA+ represented a significant improvement in modelling remoteness in Australia over the earlier Rural, Remote and Metropolitan Areas (RRMA) classification.[25] ARIA+ uses geographical information systems (GIS) to measure the road distance from all population centres to a defined hierarchy of service centres based on the principal that larger population centres provide more services and services would diminish as population size decreased. ARIA+ uses GIS raster modelling to address the modifiable areal unit problem (MAUP) that was inherent in the RRMA access model (acknowledging the use of raster data models in general does not completely overcome MAUP and users need to be aware of boundary and zoning issues that may influence interpretation).

The ARIA+ methodology measures road distances from over 12,000 population locations (henceforth referred to as localities in main text) across Australia to the nearest urban centre based on five population service centre categories, using population size as a proxy measure representing service availability at a given location. Road distance measures from localities to the five different service centre categories were standardised to a ratio score by dividing the measured distance for each locality by the Australian mean distance for each service centre category. Resulting ratio scores are then limited to a maximum of three (three times the national average) to remove the effects of extreme values from the index (termed ‘thresholding’ within the methodology) and then summed to create a standardised ARIA+ score, ranging from 0 (high accessibility) to 15 (highly remote) for each populated locality.[23] The ABS use the ARIA+ scores to classify five Remoteness Area classes: (1) Major Cities (2) Inner Regional Australia; (3) Outer Regional Australia; (4) Remote Australia; and (5) Very Remote Australia and since 2001 has been integrated as part of the Australian Census Geography (the Australian Standard Geographical Classification (ASGC) and the Australian Statistical Geography Standard (ASGS).[24] The ARIA+ methodology has been customised for specific purposes such as Pharmacy ARIA,[26] General Practitioner (GP) ARIA,[27] State-based ARIA[28] and more recently, Metro ARIA (Taylor and Lange, 2015),[29] and has been used to guide funding around workforce maldistribution (e.g., the Rural Health Multidisciplinary Training Program).[30] The ARIA+ values used in these analyses are based upon the 2011 Census of Population and Housing.

### The Cardiac Accessibility and Remoteness Index Australia (Cardiac ARIA)

In 2010, the ARIA+ methodology was applied to calculate a new service specific accessibility index which measured the distance to cardiac services across Australia.[31,32] Cardiac ARIA was calculated using the geocoded location of a comprehensive listing of cardiac services including, but not limited to, ambulance stations, hospitals and cardiac rehabilitation facilities. It has both an acute care and an ongoing management component (aftercare). Cardiac ARIA calculated road distance access from all localities to the nearest and best medical facility within 60 minutes travel for an acute cardiac event (based on ‘*The Golden Hour*’),[33] and to key services upon return to the community, the aftercare component. Acute timeframes were calculated to include time for ambulance to arrive, assess and load patient, and travel to the facility. The acute phase of the index was classified into eight integer categories based on time to different classes of medical centre: Category 1 (access to a principal referral hospital with a cardiac catheter laboratory within one hour) to Category 8 (no ambulance service or any medical faculty within three hours).[31] The aftercare index was classified into five alphabetical categories based on availability of a medical centre or doctor, retail pharmacy, cardiac rehabilitation and pathology services: Category A (all services available within one hour) to Category E (no services available within one hour). The differences and similarities between ARIA+ and Cardiac ARIA are summarised in Table 1.

**Table 1:** Differences and similarities between ARIA+ and Cardiac ARIA index methods.

ARIA+	Cardiac ARIA
Cost Distance Analysis via the •road network •cost = distance (kilometres)	Cost Distance Analysis via the •road network •cost = time
Measures access to: •5 levels of Service Centres based on the ABS Urban Centre/Locality population breakpoint categories: A. 250,000 or more B. 48,000–249,000 C. 18,000–47,999 D. 5,000–17,999 E. 1,000–4,999	Measures access to: Cardiac acute care facilities and Cardiac aftercare services
Resulting index scores are continuous, ranging from 0.00 to 15.00	For acute care, measurements are determined by ‘*The Golden Hour*’–i.e. what is the best medical facility that can be reached within an hour? For aftercare, what facilities are available within an hour’s drive?
The index can be used as a continuous index. Scores have also been categorised (most notably by the ABS for the Remoteness Areas Classification), comprising five breakpoint categories ^a^: •Major Cities: 0 to 0.2 •Inner Regional: >0.2 to 2.4 •Outer Regional: >2.4 to 5.92 •Remote: >5.92 to 10.53 •Very Remote: >10.53 to 15	Index thresholded into: •8 acute categories (1–8, ranging from *≤1 hour from category 1 hospital* to *>3 hours to any medical facility* •5 aftercare categories (A-E, ranging from *≤1 hour from GP/Nurse Clinic*, *Pharmacy*, *Cardiac rehabilitation*, *Pathology* through to *No services within 1 hour by road*) Resulting in: •19 categories when combined ^b^ (1A, 2A, 3A, 4A, 4B, 4C, 5A, 5B, 5C, 5D, 6A, 6B, 6C, 6D, 6E, 7D, 8C, 8D, 8E)
•Accessibility/remoteness calculations completed for 12,000+ individual localities then •Interpolated to 1 km x 1 km cells •Individual results can be extracted for any geographic location or area	•Analysis done on 200 m x 200 m cells for all of Australia, then •Individual results extracted for 20,000 + localities
Measures Distance: •Road distance from localities to service centres calculated in kilometres	Measures Time including: •Dispatch time of ambulance •Travel time to scene •Assessment time at scene •Travel time to hospital or clinic Different times/speeds for •Rural vs. urban dispatch •Rural vs. urban assessment •Rural vs. urban driving speed •Sealed vs. unsealed road speed
Road distance measured from locality to the edge of the nearest service centre boundaries for each of five service centre categories	Measures distance from locality centre to locality centre

^a^ Population summary by ABS Remoteness Structure [34]

^b^ Population summary by Cardiac ARIA category [31]

### Analysis: Comparison of ARIA+ and Cardiac ARIA

Both ARIA+ and Cardiac ARIA use the same basic methodology measuring cost distance along the road network to geographic locations or ‘localities’ representing population centres. ARIA+ uses urban centre and locality populated areas to derive service centres classified by size as a proxy for service provision, while Cardiac ARIA uses actual cardiac and medical services locations. ARIA+ used the ratio of the Australian mean distance to the five population centre sizes while Cardiac ARIA used time to the best possible medical service based on the ‘*golden hour*’. The average distance ratio would not provide a meaningful measure for access to medical services as the time to the best medical facility is critical for survival. Results have been presented as cross-tabulations including number and percentage to quantify the associations between characteristics of the two indices (SPSS). Pearson chi-square test for independence was used to test for association between ARIA and CARIA categories and the strength of the association was tested using Cramer’s V.[35] Map visualisations were produced using GIS (ArcGIS). All Australian localities with ARIA+ and Cardiac ARIA values were included in the analysis (n = 20,223).

## Results

To provide a means of describing the similarities and differences between the two indices, Cardiac ARIA categories (both acute and aftercare) were cross classified against ARIA+ classes. There were significant associations between ARIA+ and Cardiac ARIA acute (Table 2, χ^2^ = 25250.73, *df* = 28, p<0.001, Cramer’s V = 0.559, p<0.001) and Cardiac ARIA aftercare (Table 3, χ^2^ = 17204.38, *df* = 16, Cramer’s V = 0.461, p<0.001).

**Table 2:** Australian localities by Cardiac ARIA (acute) and ARIA+ Categories. Red shading indicates good acute cardiac services relative to the ARIA+ category. Blue shading indicates poor acute cardiac services relative to the ARIA+ category. Localities displayed in Fig 1.

Cardiac ARIA Acutecare Category	Major Cities of Australia		Inner Regional Australia		Outer Regional Australia		Remote Australia		Very Remote Australia		Total	
	No.	%	No.	%	No.	%	No.	%	No.	%	No.	%
1	2757	76.3	628	17.4	230	6.4					3615	100
2	270	21.9	887	72.0	52	4.2	23	1.9			1232	100
3	58	5.4	750	69.6	247	22.9	22	2.0			1077	100
4	28	1.4	1203	59.5	792	39.1					2023	100
5			1231	30.1	2149	52.5	532	13.0	183	4.5	4095	100
6	19	0.3	1190	17.6	3189	47.0	1360	20.1	1022	15.1	6780	100
7							4	2.2	175	97.8	179	100
8			4	0.3	24	2.0	165	13.5	1029	84.2	1222	100
Total	3132	15.5	5893	29.1	6683	33	2106	10.4	2409	11.9	20223	100

The acute phase of the index has 8 numeric categories based on time to different classes of medical centre: Category 1 (access to a principal referral hospital with a cardiac catheter laboratory within one hour) to Category 8 (no ambulance service or any medical faculty within three hours of the population location).[31]

**Table 3:** Australian localities by Cardiac ARIA (aftercare) and ARIA+ Categories. Red shading indicates good aftercare cardiac services relative to the ARIA+ category. Blue shading indicates poor aftercare cardiac services relative to the ARIA+ category. Localities displayed in Fig 2.

Cardiac ARIA Aftercare Category	Major Cities of Australia		Inner Regional Australia		Outer Regional Australia		Remote Australia		Very Remote Australia		Total	
	#	%	#	%	#	%	#	%	#	%	#	%
A	3132	23.1	5705	42.1	4397	32.4	290	2.1	39	0.3	13563	100
B			48	5.0	666	69.0	208	21.6	43	4.5	965	100
C			129	5.0	1413	55.3	719	28.1	296	11.6	2557	100
D			8	0.5	143	8.7	538	32.7	957	58.1	1646	100
E			3	0.2	64	4.3	351	23.5	1074	72.0	1492	100
Total	3132	15.5	5893	29.1	6683	33.0	2106	10.4	2409	11.9	20223	100

5 alphabetical categories based on availability of a medical centre or doctor, retail pharmacy, cardiac rehabilitation and pathology services: Category A (all services available within one hour) to Category E (no services available within one hour of the population location)[31]

The combination of the highest level of acute cardiac access (Cardiac ARIA Category 1) with the highest general ARIA+ (Major Cities) contained 2757 localities (13.6% of locations) (Table 2). The poorest acute cardiac access (Cardiac ARIA Category 8) and Very Remote Australia was shared in 1029 localities (5.1% of locations). Cardiac ARIA Category 6 (>3 hours access to any acute cardiac care) was the only Cardiac ARIA category represented in all five ARIA+ categories (Table 2). Within this category (Cardiac ARIA Category 6) was the combination with the highest number of observations (3189 localities, 15.8% of total), which was in Outer Regional Australia.

Aftercare services were geographically well distributed and accorded well with the ARIA+ classifications (Table 3). Approximately three quarters of localities in Australia were classed as Cardiac ARIA Category A, access to all services needed for cardiac primary and secondary prevention (Table 3). Localities in Major Cities, Inner Regional Australia, and Outer Regional Australia accounted for almost 98% of this category. Outer Regional Australia with Cardiac ARIA Category C (n = 1413), and Very Remote Australia with Cardiac ARIA Category E (n = 1074) were the only other two combinations with greater than 1000 observations.

**Fig 1:**
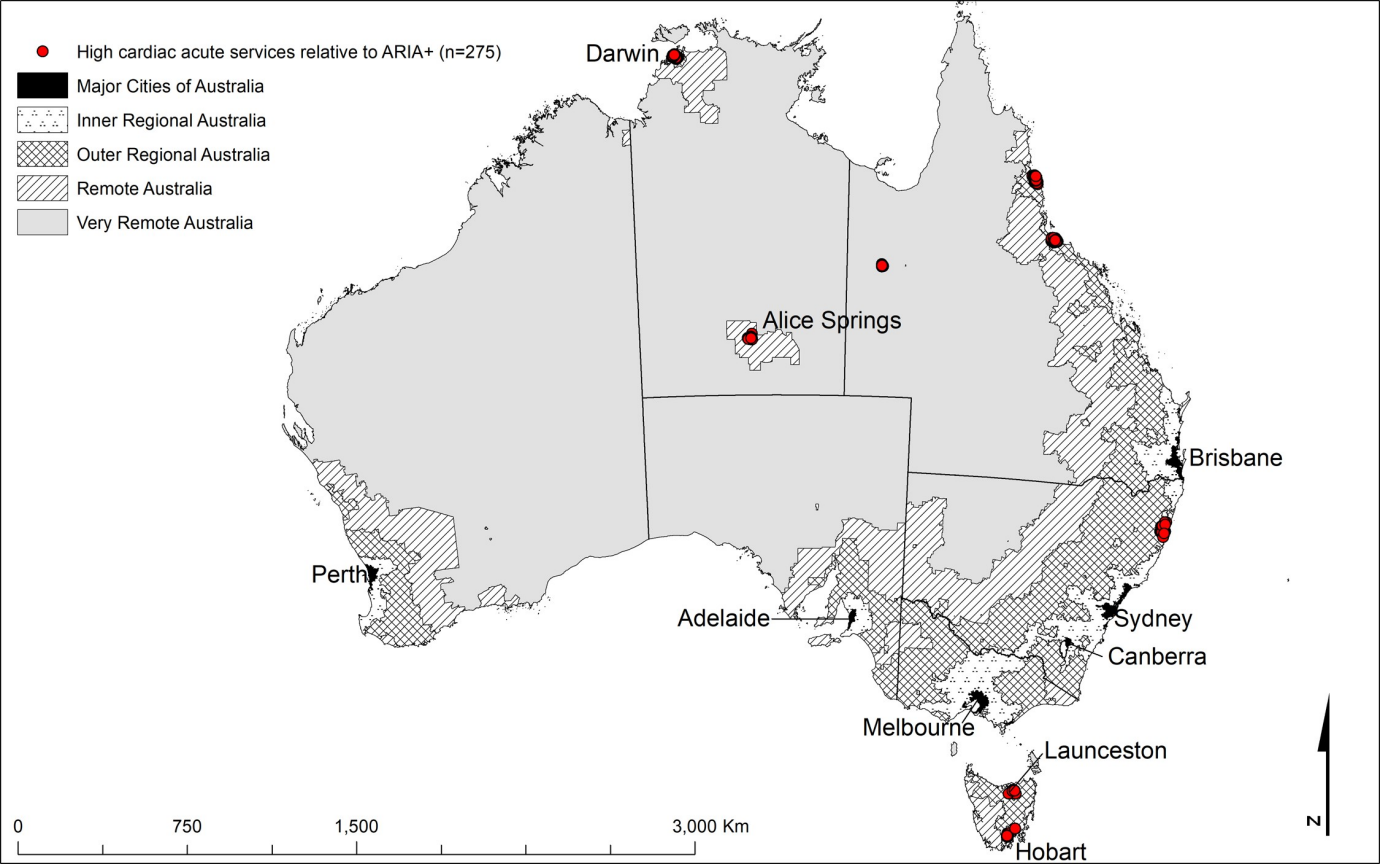
The geographical locations of the shaded cells from Table 2 highlighting the main discrepancies between Cardiac ARIA (acute) and the ARIA+ remoteness structure[24] (high acute cardiac services relative to ARIA+) (n = 275).

**Fig 2:**
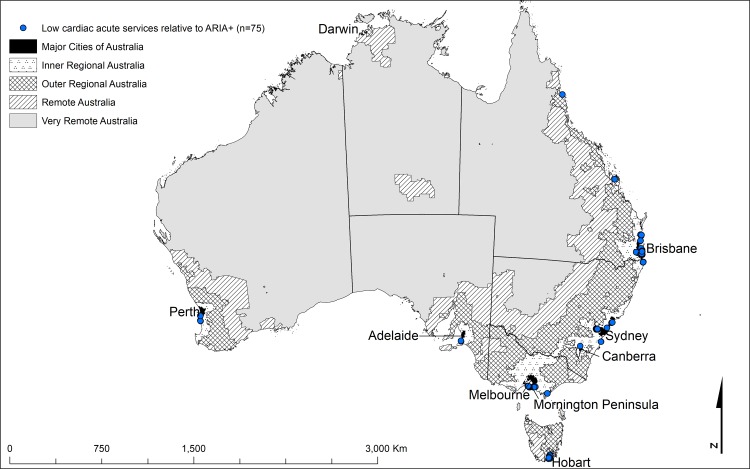
The geographical locations of the shaded cells from Table 3 highlighting the main discrepancies between Cardiac ARIA (after) and the ARIA+ remoteness structure[24] (low acute cardiac services relative to ARIA+) (n = 75).

**Fig 3:**
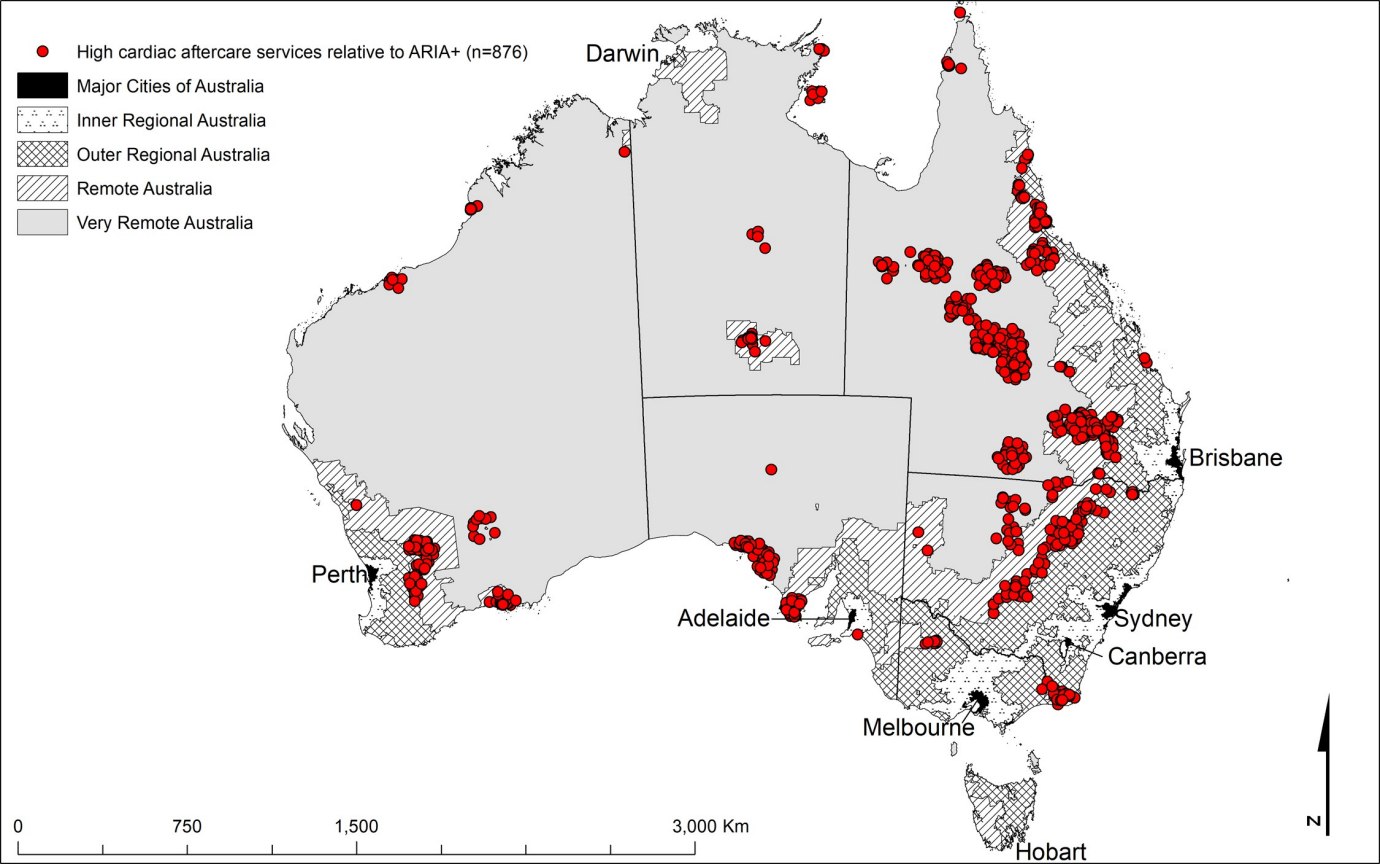
The geographical locations of the shaded cells from Table 2 highlighting the main discrepancies between Cardiac ARIA (acute) and the ARIA+ remoteness structure[24] (high aftercare cardiac services relative to ARIA+) (n = 876).

**Fig 4:**
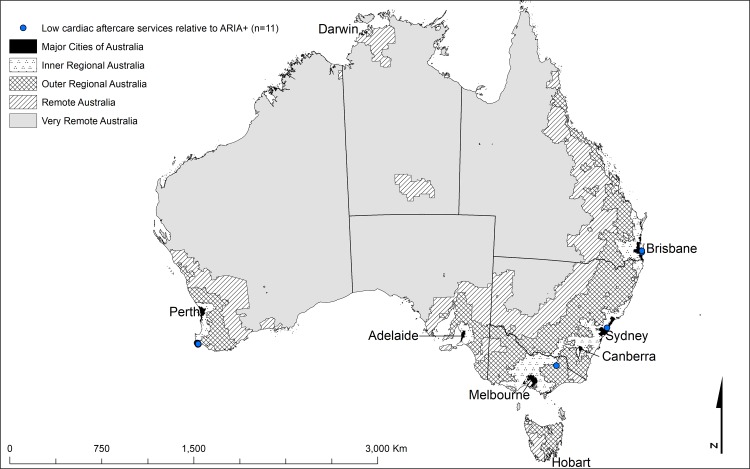
The geographical locations of the shaded cells from Table 3 highlighting the main discrepancies between Cardiac ARIA (aftercare) and the ARIA+ remoteness structure[24] (low aftercare cardiac services relative to ARIA+) (n = 11).

The important aspect of this comparison is where the two indices differ. The shaded cells in Tables 2 and 3 highlight deviations from the areas of agreement and identify locations with the highest disagreement. Cardiac ARIA and ARIA+ discrepancies occurred in clusters indicating differences that were not random (Figs 1–4). The figures identify localities where cardiac services (both acute, Fig 2, and aftercare, Fig 4) were low relative to the ARIA+ category (less cardiac service than the population size would indicate, shown in blue). Also identified are localities with a better level of cardiac services than indicated by the population size (good cardiac services relative to the ARIA+ category, shown in red, Figs 1 and 3). Good acute care relative to remoteness typically occurred in the larger centres outside Major Cities of Australia (e.g., localities surrounding Darwin, Alice Springs, Launceston, and Hobart) (indicated by red in Fig 1). In contrast, localities with relatively poor levels of acute care access relative to remoteness tended to be on the outskirts of Major Cities of Australia where local geography prevented access within the ‘*golden hour’* (e.g., the furthest extent of the Mornington Peninsula south of Melbourne, in localities such as Portsea and Sorrento, indicated by blue markers in Fig 2). The other main contributor to this category were localities situated on islands (e.g., numerous islands close to Brisbane classed as Inner Regional Australia yet classed as 8E when applying Cardiac ARIA, indicated by blue in Fig 2).

There were many more examples of highlighted discrepancies between ARIA+ and Cardiac ARIA (aftercare) (Table 3 and Figs 3 and 4) relative to Cardiac ARIA (acute) (Table 2 and Figs 1 and 2). One of the main drivers of the patterns of localities with good aftercare access relative to general access tended to be localities proximal to a centre with services, but located in a more remote ARIA+ category (indicated by red in Table 3, n = 876, and Fig 3). In comparison, there were far fewer discrepancies where general access was good but cardiac aftercare was relatively poor (indicated by blue in Table 3, n = 11 and Fig 4).

## Discussion

While there is evidence within the literature that compares health systems,[36] and health indices,[37] to our knowledge this the first comparison of geographical access index models for the purpose of improving health policy and health service provision at the continental level. Recent research includes direct comparisons at smaller spatial extents,[38] but comparisons at much larger areas appear to be lacking. Furthermore, more localised approaches tend to focus on model performance,[38,39] or describing variations in patterns of access as the geographical extent becomes larger.[40] The aim of this research was to describe the underlying conceptual similarities and differences between a more general access model (ARIA+) and the service specific Cardiac ARIA index. The first step was to identify locations where the levels of accessibility substantially differ across models–this will inform policy direct influenced by the two models. Secondly, we highlight the appropriate use of geographically modelled data for decision making–a finding that will be generalisable to many different scenarios.

This study demonstrated broad agreement between the two access index models. This result is not unexpected given the strength and similar underlying methodology, coupled with the highly urbanised nature of Australia (71% of Australians live in major cities).[41] The influence of Australia’s population distribution and resultant association with services confers high agreement between ARIA+ and Cardiac ARIA in metropolitan areas and remote and very remote areas.

Nonetheless, this study has highlighted some important systematic differences between ARIA+ and Cardiac ARIA that are largely attributable to the different purposes for which they were designed. These differences are most evident in the peri-urban areas of the major capital cities, and around the more remote regional centres. The variation identified in this paper emphasises the importance of the appropriate use of models. These findings substantiate the need and value of using an index that utilises precise service locations (i.e., Cardiac ARIA) to inform and optimise cardiac health service provision for the best cardiac health outcomes. The same principal applies to the provision of other health services. The comparison with ARIA+ serves to highlight that using a general measure of remoteness based on *distance to population centres* does not reflect accessibility to cardiac services which is based upon *distance to services* and is *time* critical. If ARIA+ was used for planning cardiac services it would result in some areas having suboptimal cardiac health service provision, suboptimal cardiac health outcomes, and inefficient allocation of health funding.

Despite the underlying similarities in methodology, these two models measure different aspects of access and have some important differences in orientation. Both indices make a valuable contribution to understanding geographic accessibility, although there is a need to more clearly outline the reasons to use one index in preference to the other for specific applications. While the two indices share the common GISCA/Hugo Centre for Migration and Population Research methodological approach of measuring and quantifying access, the fundamental differences between the two indices is their specificity of service location and the application of time, in particular ‘*the golden hour*’. These results in the differences in the spatial representation of access between the two indices and underpins the importance of understanding the reasons for these differences and the impact for policy and services planning.

### Implications for applied use and future direction

One of the overarching objectives of access modelling at the national level is to provide policy-makers and planners with a tool to assist with decision making and funding allocation. Each accessibility model is also subject to a set of computations which have implications for real world applications and interpretations (e.g., Cardiac ARIA was measured to point localities whereas ARIA+ was measured to service centre boundaries). This is particularly important when these differences manifest in a systematic manner. While it is tempting to say that understanding these nuances is the job of those using models to aid strategic decision making, it is equally incumbent upon health geographers to ensure such tools have clearly defined caveats to mitigate against erroneous interpretation. By quantifying and explaining their origins, this paper has gone some way to redressing the potential suboptimal application of modelling in the planning process.

There is a need for service-based indices to be updated regularly as localised changes can have a substantial impact on the index values, not to mention the real-world implications for service levels (e.g., hospital, ambulance station, or aftercare facility opening or closure).

## Conclusions

The contribution this study will make to policy and practice is to demonstrate that for health service planning and delivery, specific targeted access models are required. While broadly comparable, ARIA+ and Cardiac ARIA are not interchangeable, and systematic differences are apparent which can be attributed largely to the underlying specificity of the Cardiac ARIA model versus the general nature of the ARIA+ model–a principal that extends beyond the provision of cardiac services. Therefore, the reasoning behind the differences highlighted will be generalisable to any comparison between general and service-specific access models. The use of the correct tool can potentially lead to better policy decisions, efficient deployment of resources, and targeted service provision. Additionally, there is an ongoing role for geographers to be transparent when communicating the advantages and limitations of such tools.
