# Correction: Comparison of general and cardiac care-specific indices of spatial access in Australia

**DOI:** 10.1371/journal.pone.0221465

**Published:** 2019-08-15

**Authors:** Vincent Lawrence Versace, Neil T. Coffee, Julie Franzon, Dorothy Turner, Jarrod Lange, Danielle Taylor, Robyn Clark

There are errors in the captions for Tables 2 and 3, Figs 2 and 3. Please see the complete, correct Tables 2 and 3, Figs 2 and 3 captions here.

**Table 2:** Australian localities by Cardiac ARIA (acute) and ARIA+ Categories. Red shading indicates good acute cardiac services relative to the ARIA+ category. Blue shading indicates poor acute cardiac services relative to the ARIA+ category. Localities displayed in Fig 1 and Fig 2.

Cardiac ARIA Acutecare Category	Major Cities of Australia		Inner Regional Australia		Outer Regional Australia		Remote Australia		Very Remote Australia		Total	
	No.	%	No.	%	No.	%	No.	%	No.	%	No.	%
1	2757	76.3	628	17.4	230	6.4					3615	100
2	270	21.9	887	72.0	52	4.2	23	1.9			1232	100
3	58	5.4	750	69.6	247	22.9	22	2.0			1077	100
4	28	1.4	1203	59.5	792	39.1					2023	100
5			1231	30.1	2149	52.5	532	13.0	183	4.5	4095	100
6	19	0.3	1190	17.6	3189	47.0	1360	20.1	1022	15.1	6780	100
7							4	2.2	175	97.8	179	100
8			4	0.3	24	2.0	165	13.5	1029	84.2	1222	100
Total	3132	15.5	5893	29.1	6683	33	2106	10.4	2409	11.9	20223	100

The acute phase of the index has 8 numeric categories based on time to different classes of medical centre: Category 1 (access to a principal referral hospital with a cardiac catheter laboratory within one hour) to Category 8 (no ambulance service or any medical faculty within three hours of the population location).[31]

**Table 3:** Australian localities by Cardiac ARIA (aftercare) and ARIA+ Categories. Red shading indicates good aftercare cardiac services relative to the ARIA+ category. Blue shading indicates poor aftercare cardiac services relative to the ARIA+ category. Localities displayed in Fig 3 and Fig 4.

Cardiac ARIA Aftercare Category	Major Cities of Australia		Inner Regional Australia		Outer Regional Australia		Remote Australia		Very Remote Australia		Total	
	#	%	#	%	#	%	#	%	#	%	#	%
A	3132	23.1	5705	42.1	4397	32.4	290	2.1	39	0.3	13563	100
B			48	5.0	666	69.0	208	21.6	43	4.5	965	100
C			129	5.0	1413	55.3	719	28.1	296	11.6	2557	100
D			8	0.5	143	8.7	538	32.7	957	58.1	1646	100
E			3	0.2	64	4.3	351	23.5	1074	72.0	1492	100
Total	3132	15.5	5893	29.1	6683	33.0	2106	10.4	2409	11.9	20223	100

5 alphabetical categories based on availability of a medical centre or doctor, retail pharmacy, cardiac rehabilitation and pathology services: Category A (all services available within one hour) to Category E (no services available within one hour of the population location)[31].

**Fig 2:**
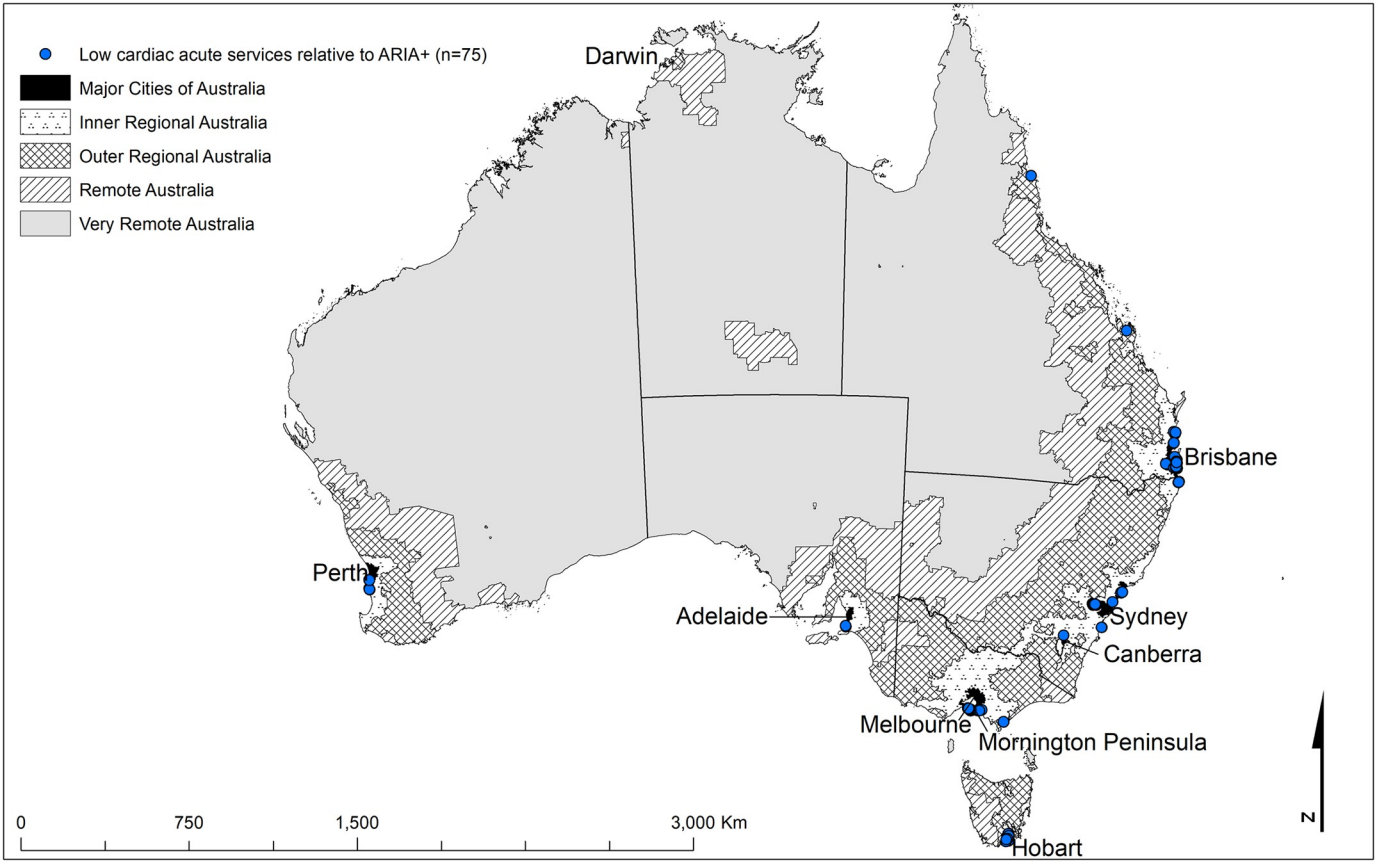
The geographical locations of the shaded cells from Table 2 highlighting the main discrepancies between Cardiac ARIA (after) and the ARIA+ remoteness structure [24] (low acute cardiac services relative to ARIA+) (n = 75).

**Fig 3:**
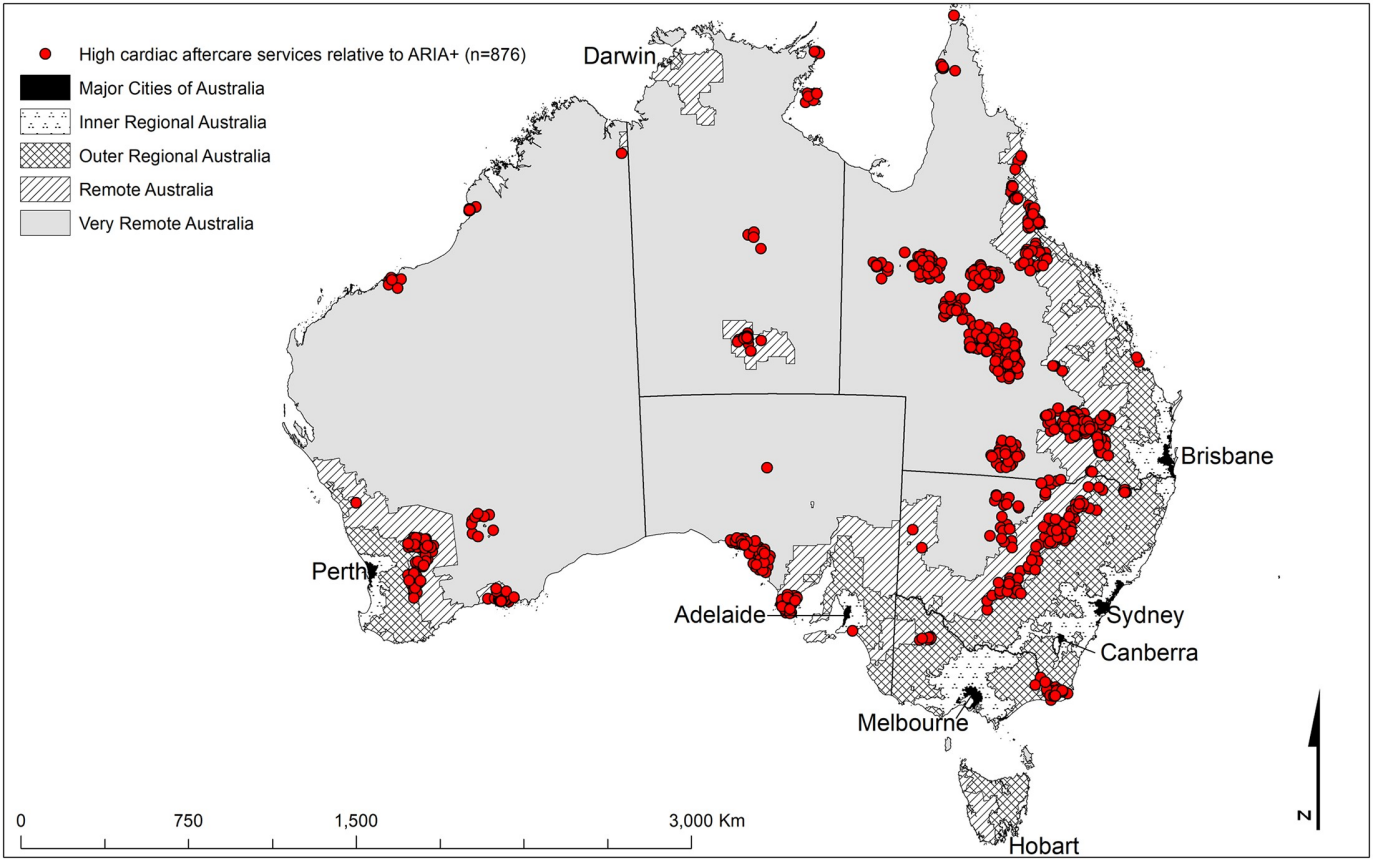
The geographical locations of the shaded cells from Table 3 highlighting the main discrepancies between Cardiac ARIA (acute) and the ARIA+ remoteness structure [24] (high aftercare cardiac services relative to ARIA+) (n = 876).
